# Anaemia at mid-pregnancy is associated with prehypertension in late pregnancy among urban women

**DOI:** 10.4102/hsag.v29i0.2610

**Published:** 2024-06-29

**Authors:** Caylin Goodchild, Elizabeth A. Symington, Jeannine Baumgartner, Lizelle Zandberg, Amy J. Wise, Cornelius M. Smuts, Linda Malan

**Affiliations:** 1 Centre of Excellence for Nutrition, Faculty of Health Sciences, North-West University, Potchefstroom, South Africa; 2 Department of Life and Consumer Sciences, School of Agriculture and Life Sciences, University of South Africa, Johannesburg, South Africa; 3 Department of Nutritional Sciences, King’s College London, London, United Kingdom; 4 Department of Obstetrics and Gynaecology, Faculty of Health Sciences, University of Witwatersrand, Johannesburg, South Africa; 5 Empilweni Services and Research Unit, University of the Witwatersrand, Johannesburg, South Africa

**Keywords:** anaemia, prehypertension, blood pressure, fasting glucose, gestation, glucose tolerance, iron supplementation, pregnancy

## Abstract

**Background:**

Antenatal iron deficiency and anaemia are associated with gestational hypertension and diabetes mellitus, but so are elevated iron stores and haemoglobin. In South Africa, pregnant women receive routine iron supplementation regardless of iron status.

**Aim:**

This study aimed to assess associations of antenatal iron status and anaemia with blood pressure in pregnant women in urban South Africa. Secondary to this, associations with heart rate, fasting glucose and glucose tolerance were also investigated.

**Setting:**

Johannesburg, South Africa.

**Methods:**

A total of 250 pregnant women, aged 27 (24–32) years, were recruited using consecutive sampling. The authors measured biomarkers of iron status and anaemia at < 18 and ± 22 weeks’, blood pressure and heart rate at ± 36 weeks’, and fasting glucose and glucose tolerance between 24 and 28 weeks’ gestation. Associations were determined using multivariable regression models adjusted for confounders.

**Results:**

The odds of prehypertension in late pregnancy among women with anaemia at ± 22 weeks’ gestation were three times higher than among women without anaemia (odds ratio [OR]: 3.01, 95% confidence interval [CI]: 1.22, 7.42). Participants with anaemia at ± 22 weeks’ gestation had 2.15 times higher odds of having elevated mean arterial pressure than women without anaemia (OR: 2.15, 95% CI: 1.01, 4.60).

**Conclusion:**

Anaemia at mid-pregnancy could be a predictor of hypertensive disorders in pregnancy. The cause of antenatal anaemia may need further investigation apart from iron deficiency. The effective management of anaemia in pregnant women living in urban South Africa remains a challenge.

**Contribution:**

This study provides evidence about the health impact of pregnant women regarding antenatal supplementation practices in South Africa.

## Introduction

Anaemia is a severe global public health nutrition problem (World Health Organization [WHO] 2023). According to the South Africa Demographic and Health Survey, the anaemia prevalence among women 15–49 years of age was 33%, indicating a moderate public health problem in South Africa (National Department of Health [NDoH] et al. 2019). National anaemia data in pregnant women do not exist; however, smaller studies reported the anaemia prevalence of 29% to 43% in South African pregnant women (Symington et al. 2019; Tunkyi & Moodley 2018). A major contributing factor to anaemia is iron deficiency (ID). Ten per cent of women of reproductive age had iron deficiency anaemia (IDA) in the 2012 South African national survey (Shisana et al. 2013).

Previously, the WHO recommended daily oral iron supplementation with 30 mg to 60 mg of elemental iron for all pregnant women to prevent IDA (WHO 2016). More recent recommendations are multiple micronutrient supplementation (MMS) containing 13 to 15 micronutrients, including 30 mg of elemental iron (WHO 2020). In South Africa, routine iron supplementation (200 mg ferrous sulphate equal to ± 65 mg elemental iron daily) is provided to all pregnant women to prevent anaemia (DoH 2016). However, in certain populations, iron absorption is limited (Cappellini, Musallam and Taher 2020) and routine supplementation may not improve iron status and decrease anaemia through the course of pregnancy, as was the case in this cohort (Symington et al. 2019).

Both low and elevated haemoglobin (Hb) concentrations have been associated with maternal morbidities displaying a U-shaped association (Young et al. 2019). Common maternal morbidities associated with anaemia include hypertension, preeclampsia and sepsis, while gestational diabetes mellitus (GDM) is less common (Wang et al. 2018; Young et al. 2019). On the other hand, there is evidence of a higher Hb concentration in the second trimester placing women at risk for preeclampsia, preterm birth and low birth weight (Çakmak et al. 2018). Hypertensive disorders of pregnancy (HDP) are the most common direct cause of maternal mortality and account for 18% of all maternal deaths in South Africa (Moodley et al. 2019). Gestational hypertension is typically diagnosed after 20 weeks of pregnancy or close to delivery (CDC 2021) and mean arterial pressure (MAP) is commonly used in late pregnancy to screen for preeclampsia (Panaitescu et al. 2018). If not identified and treated, hypertension can lead to an increased risk of maternal stroke, lower birth weight and increased risk of neonatal intensive care (Webster et al. 2019). Although not of clinical significance on its own, prehypertension during late pregnancy may lead to gestational hypertension and subsequently to more serious clinical outcomes (Rosner et al. 2019).

Low Hb has also been associated with increased heart rate (Metivier et al. 2000). Generally during pregnancy, heart rate increases by 10–20 beats per minute (bmp); however, anaemia has been associated with an exacerbated increase. Tachycardia during pregnancy may lead to an increased risk of cardiac disease and maternal morbidity (Coad & Frise 2021).

Gestational diabetes mellitus is one of the leading causes of morbidity and mortality for mothers and infants worldwide, with prevalence in South Africa ranging from 1.6% to 25.8% (Dias et al. 2019). Gestational diabetes mellitus is an independent risk factor for the future development of type 2 diabetes mellitus (T2DM), cardiovascular morbidity, metabolic syndrome, malignancies, as well as ophthalmic, psychiatric and renal disease in the mother as well as T2DM, obesity, poorer neurodevelopmental outcome and ophthalmic disease in the child (Farahvar, Walfisch & Sheiner 2019).

Due to the findings that both low and elevated Hb concentrations have been associated with maternal morbidities, the primary objective of this study was to determine the association of iron status and anaemia at early (< 18 weeks) and mid-pregnancy (± 22 weeks) with prehypertension in late pregnancy (± 36 weeks’ gestation). Secondary objectives were to determine associations with blood pressure (systolic, diastolic and MAP), heart rate, fasting glucose and glucose tolerance and describe the incidence of hypertension, prehypertension and GDM in women residing in urban Johannesburg, South Africa.

## Methods

### Study design and site

This study formed part of the prospective cohort study *Nutrition during Pregnancy and Early Development* (NuPED) conducted in Johannesburg, South Africa. The study protocol was published previously (Symington et al. 2018). Pregnant women were recruited between March 2016 and November 2017 at primary healthcare clinics in Johannesburg. Non-probability sampling following a consecutive sampling technique was used; therefore, all pregnant women at the recruitment sites formed part of the sample if they met the eligibility criteria, arrived at the study site on their booked date and signed the informed consent.

### Eligibility criteria

Women were eligible if they were aged 18–39 years, < 18 weeks pregnant with singleton pregnancies, proficient in local languages, born in South Africa or neighbouring countries and had been residing in Johannesburg for at least 12 months. Women were excluded if they reported using illicit drugs, were smoking or had previously been diagnosed with a non-communicable disease (namely diabetes, renal disease, high cholesterol and hypertension), an infectious disease (namely tuberculosis and hepatitis) or a serious illness (namely cancer, lupus or psychosis). Due to South Africa’s high prevalence of HIV infection, women who were living with HIV were included in the study. The eligible women who agreed to participate were followed up at the antenatal clinic of an academic hospital until June 2018.

### Outcome measurements

The primary outcome measure was prehypertension in late pregnancy, with secondary outcome measures being systolic blood pressure (SBP) and diastolic blood pressure (DBP), MAP and heart rate in late pregnancy (± 36 weeks’ gestation) as well as fasting glucose and glucose tolerance in mid-pregnancy. Blood pressure and heart rate were also obtained at enrolment and at ± 22 weeks’ gestation to describe the blood pressure changes with the progression of pregnancy. Blood pressure and heart rate were taken twice at each time point by trained fieldworkers using calibrated equipment (Omron M3W upper arm blood pressure monitor, OMRON Healthcare, Kyoto, Japan) (Takahashi 2012). The second measurement for blood pressure and heart rate was used for analysis. Prehypertension was diagnosed if SBP was ≥ 120 mmHg and/or if DBP was ≥ 80 mmHg (Cao et al. 2020). Hypertension was diagnosed if SBP was ≥ 140 mmHg and/or if DBP was ≥ 90 mmHg. Mean arterial pressure was calculated using the following formula: DBP + 1/3(SBP – DBP). Elevated MAP (eMAP) during pregnancy was defined as MAP > 86 mmHg (Women’s Health and Education Centre 2009).

A 2 h oral glucose tolerance test (OGTT) with 75 g glucose was conducted between 24- and 28-weeks of gestation according to the Society for Endocrinology, Metabolism and Diabetes of South Africa (SEMDSA) guidelines (Du Plessis 2018). Fasting glucose was obtained by finger prick before the oral glucose was consumed. Glucose tolerance was assessed by determining plasma glucose from a venous blood sample collected in potassium oxalate and sodium fluoride-containing vacutainers (grey top tubes) 2 h post glucose consumption. Gestational diabetes mellitus was diagnosed if fasting plasma glucose was ≥ 7.0 mmol/L and/or if plasma glucose after 2 h was ≥ 11.1 mmol/L (Du Plessis 2018).

### Exposure measurements

Biomarkers of iron status, including ferritin and soluble transferrin receptor (sTfR) and Hb concentrations, were measured at enrolment and mid-pregnancy (< 18 and ± 22 weeks of gestation, respectively). Maternal venous blood was drawn into lithium heparin-coated evacuated tubes. The serum was separated within 1 h after blood was drawn and stored at −20 °C for a maximum of 14 days until transportation for storage at –80 °C until analysis. Ferritin, sTfR and inflammatory markers (α1-acid glycoprotein [AGP] and C-reactive protein [CRP]) concentrations were determined using the Q-Plex Human Micronutrient Array (7-plex, Quansys Bioscience, Logan, UT, United States [US]) (Brindle et al. 2017). The inflammatory markers were used to correct ferritin for inflammation (Thurnham, Northrop-Clewes and Knowles 2015). Iron deficiency was defined as adjusted ferritin < 15 µg/L (WHO 2011a). Iron deficiency erythropoiesis (IDE) was defined as sTfR > 8.3 mg/L (Silubonde et al. 2023).

Hb concentrations were determined in venous whole blood samples (20 µL) using calibrated HemoCue metres (Hb 201+, Ängelholm, Sweden). Due to Johannesburg’s location being 1753 m above sea level (Topographic Maps 2001), Hb values were adjusted for altitude (WHO 2011b). Anaemia was defined as Hb < 11 g/dL at < 18 weeks of gestation and Hb < 10.5 g/dL at mid-pregnancy based on cut-offs per trimester (South Australian Maternal & Neonatal Community 2016). In addition, for the purpose of comparability, the prevalence of anaemia was also reported according to the WHO (2017) Hb cut-off (< 11 g/dL) at mid- and late pregnancy.

### Covariates

Maternal age and living standards measurements (LSM), indicating the socio-economic status of the women, were obtained by use of a questionnaire conducted by an interviewer at baseline (Truter 2007). These measures divide the population into 10 LSM groups, where one is the lowest living standard level and 10 is the highest level. This tool uses criteria such as degree of urbanisation, as well as ownership of cars and appliances. Socio-economic status is associated with both iron status and high blood pressure (Manikam 2021; Robinson et al. 2021). High maternal body mass index (BMI) is a known risk factor for poor iron status, hypertension and increased heart rate (Du Toit, Schutte and Mels 2020; Köchli, Schutte & Kruger 2020; Silubonde et al. 2023) in African women. Body mass index at enrolment was thus considered a covariate and calculated as weight (kg) at enrolment, that is < 18 weeks’ gestation, divided by height (m) squared, unadjusted for gestational age. According to Inskip et al. (2020), a measured weight in early pregnancy, which the authors used, provides a more precise assessment of pre-pregnancy weight than recalled weight. Maternal HIV status was obtained from medical records and was updated again at birth, as anaemia is a common complication of HIV (Cao et al. 2022). Maternal CRP, an indicator of inflammation, was also measured at baseline and throughout pregnancy. Inflammation is positively associated with increased blood pressure (Köchli et al. 2020). HIV infection as well as overweight and obesity can both contribute to a more inflammatory state. This could also result in increased hepcidin concentrations, which reduces iron absorption and the release of iron from hepatic stores (Bah et al. 2019). Gestational age was determined using foetal ultrasonography (Symington et al. 2018). Gestational age at enrolment varied between 6 and < 18 weeks, leading to a wide variance in exposure to antenatal iron supplementation. Aspirin is recommended to women at risk for preeclampsia; therefore, aspirin prescription at mid-pregnancy (22 weeks’ gestation) was attained from medical records and included as a covariate (Davidson et al. 2021). To assess how anaemia and iron status may be associated with pregnancy-induced high blood pressure, an additional model correcting for blood pressure at ± 22 weeks was tested. These covariates were included in the regression models determining the associations of antenatal iron status and anaemia with blood pressure, fasting glucose and glucose tolerance in pregnant women residing in urban South Africa.

### Statistical analysis and data management

The post hoc power calculation was based on multiple linear regression analysis (fixed model, single regression coefficient). A sample size of 200 provided 80% power to detect a small effect size *F*^2^ of 0.08 with a probability of error (alpha) of 5% when including eight predictors. Data processing and statistical analysis of data were performed using SPSS version 27 (SPSS Inc, Chicago, IL, US). Raw data were captured in Microsoft Access (Microsoft Corporation, Washington, US) and 20% of all captured data were randomly checked for correctness. Data were tested for outliers and normality by means of Q-Q plots, histograms and Shapiro–Wilk tests. Normally distributed data are expressed as means ± s.d.; non-normally distributed data are expressed as medians (interquartile range [IQR]). Longitudinal analysis of continuous blood pressure and GDM biomarkers over three time points in pregnancy was performed with the repeated measures ANOVA with Bonferroni corrections for between-group analyses. Sphericity was assumed when *p* > 0.05 with Mauchly’s test. Greenhouse–Geisser was used when sphericity was violated. Categorical associations were analysed with McNemar’s test for dependant data. Associations of maternal iron status and anaemia at early (< 18 weeks’ gestation) and mid-pregnancy (± 22 weeks’ gestation) with SBP, DPB, MAP and heart rate in late pregnancy were assessed with multivariable logistic and linear regression. Odds ratio (Exp B) and B-values, respectively, and 95% confidence intervals were reported. Model 1 was adjusted for maternal age and LSM (socio-economic status); model 2 was adjusted additionally for HIV status, maternal BMI at enrolment, and aspirin prescription during mid-pregnancy, gestational age and CRP; and model 3 was adjusted additionally for blood pressure at ± 22 weeks’ gestation. To investigate if the relationship between iron status and anaemia with blood pressure is U-shaped, univariate comparisons were carried out between quartiles of each iron biomarker (Hb, ferritin and sTfR) at early and mid-pregnancy with SBP and DBP in late pregnancy. This was adjusted for maternal age, LSM (socio-economic status), HIV status, maternal BMI at enrolment, aspirin prescription at mid-pregnancy, gestational age, CRP and blood pressure at ± 22 weeks’ gestation. Associations of iron status biomarkers with fasting glucose and glucose tolerance were assessed with multivariable linear regression, B-values and 95% confidence intervals. Model 1 was unadjusted (crude); model 2 adjusted for maternal age and LSM (socio-economic status), HIV status, maternal BMI at enrolment and gestational age; and model 3 adjusted additionally for CRP. *p* < 0.05 was considered significant. Dichotomous analyses were not performed as too few participants had values above the cut-off for prediabetes (fasting glucose 5.6 mmol/L–6.9 mmol/L), *n* = 2 or diabetes (≥ 7.0 mmol/L), *n* = 1 or impaired glucose tolerance with the OGTT (glucose 7.8 mmol/L–11.0 mmol/L), *n* = 3 (WHO 1985).

### Ethical considerations

Ethical approval to conduct the study was obtained from the Health Research Ethics Committee at a large North-western public university (NWU-00186-15-A1) and the Human Research Ethics Committee at a large public university in the Witwatersrand area in Johannesburg (M150968). Written informed consent was obtained from the participants of the study. Data were kept confidential by using an anonymised coding process.

## Results

### Participant characteristics

Characteristics of the total sample of pregnant women (*n* = 250) at enrolment (< 18 weeks of gestation), as well as by blood pressure status in late pregnancy, are shown in Table 1. Eighty-eight per cent of women were of black-African descent. At enrolment, the women’s median age was 27 (24, 32) years and median gestational age was 14 (12, 16) weeks. Most women completed their education in Grades 11 and 12 (58%), with 23% continuing their post-school education. Fifty-nine per cent of women had a medium LSM score indicating a middle-class living standard. Thirty per cent were nulliparous. The median BMI at enrolment was 26.3 (23.0, 30.6) kg/m^2^, with 33% of women being overweight and 28% being obese. A total of 60% of women had an elevated CRP and 26% were HIV positive.

**TABLE 1:** Characteristics of pregnant women at enrolment (< 18 weeks of gestation) and by blood pressure in late pregnancy.

Characteristics	At enrolment (< 18 weeks) (*n* = 250)				Late pregnancy blood pressure status (± 36 weeks)															
					SBP ≥ 120 mmHg (*n* = 24)				SBP < 120 mmHg (*n* = 172)				DBP ≥ 80 mmHg (*n* = 17)				DBP < 80 mmHg (*n* = 179)			
	Median	CI	*n*	%	Median	CI	*n*	%	Median	CI	*n*	%	Median	CI	*n*	%	Median	CI	*n*	%
Age (years)	27	27	-	-	31	27–33	-	-	27	26–29	-	-	30	26–33	-	-	27	26–29	-	-
Gestational age at enrolment (weeks)	14	14	-	-	15	13–16	-	-	14	14–15	-	-	15	14–17	-	-	14	14–15	-	-
**BMI (kg/m^2^)^1^**	26.3	26.3	-	-	29.6	26.4–35.4	-	-	25.9	25.0–27.5	-	-	29.8	26.4–31.7	-	-	25.9	25.1–27.5	-	-
Underweight	-	-	8	3	-	-	0	0	-	-	7	4	-	-	-	-	-	-	7	4
Normal weight	-	-	89	36	-	-	5	21	-	-	66	39	-	-	4	24	-	-	67	38
Overweight	-	-	81	33	-	-	8	33	-	-	54	32	-	-	6	35	-	-	56	32
Obese	-	-	71	28	-	-	11	46	-	-	44	26	-	-	7	41	-	-	48	27
**Ethnicity**																				
Black African	-	-	219	88	-	-	21	88	-	-	151	88	-	-	16	94	-	-	156	87
Mixed ancestry	-	-	28	11	-	-	3	13	-	-	20	12	-	-	1	6	-	-	22	12
White	-	-	1	< 1	-	-	0	0	-	-	0	0	-	-	-	-	-	-	-	-
Indian	-	-	1	< 1	-	-	0	0	-	-	1	1	-	-	-	-	-	-	1	1
**LSM**																				
Low (1–4)	-	-	17	7	-	-	1	4	-	-	11	6	-	-	2	12	-	-	10	6
Medium (5–7)	-	-	148	59	-	-	12	50	-	-	106	62	-	-	7	41	-	-	111	62
High (8–10)	-	-	85	34	-	-	11	46	-	-	55	32	-	-	8	47	-	-	58	32
**Highest level of education**																				
Primary school	-	-	9	4	-	-	0	0	-	-	9	5	-	-	-	-	-	-	9	5
Grade 8 – 10	-	-	37	15	-	-	7	29	-	-	18	11	-	-	5	29	-	-	20	11
Grade 11 – 12	-	-	145	58	-	-	11	46	-	-	106	62	-	-	8	47	-	-	109	61
Post-school	-	-	58	23	-	-	6	25	-	-	39	23	-	-	4	24	-	-	41	23
**Parity**																				
Nulliparous	-	-	74	30	-	-	4	17	-	-	52	30	-	-	3	18	-	-	53	30
Primiparous	-	-	88	35	-	-	9	38	-	-	63	37	-	-	5	29	-	-	67	37
Multiparous	-	-	88	35	-	-	11	46	-	-	57	33	-	-	9	53	-	-	59	33
**HIV status**																				
Positive	-	-	64	26	-	-	7	29	-	-	42	24	-	-	5	29	-	-	44	25
**Iron status**																				
Hb (g/dL)	11.7	10.8–12.7	-	-	11.6	9.1–12.5	-	-	11.7	11.4–11.9	-	-	10.5	8.8–12.0	-	-	11.7	11.5–11.9	-	-
Fer (μg/L)	47.8	20.8–100.8	-	-	41.5	26.5–58.5	-	-	48.2	37.1–60.9	-	-	44.9	30.5–55.7	-	-	47.8	35.7–58.8	-	-
sTfR (mg/L)	4.8	3.7–6.6	-	-	4.7	4.0–5.8	-	-	4.9	4.5–5.3	-	-	4.6	4.0–6.2	-	-	4.9	4.5–5.3	-	-
Anaemia (Hb < 11g/dL)	-	-	70	29	-	-	9	38	-	-	49	30	-	-	9	53	-	-	49	28
Anaemia (Hb < 10.5g/dL)	-	-	43	18	-	-	8	33	-	-	26	16	-	-	8	47	-	-	26	15
ID (Fer < 15μg/L)	-	-	37	15	-	-	2	8	-	-	27	15	-	-	1	6	-	-	26	15
IDE (sTfR > 8.3mg/L)	-	-	38	15	-	-	4	17	-	-	27	16	-	-	4	24	-	-	27	15
**Inflammatory status**																				
CRP (mg/L)	6.5	3.1–14.1	-	-	12.1	4.8–18.7	-	-	6.9	5.5–9.2	-	-	7.7	4.2–14.1	-	-	7.0	5.7–10.4	-	-
Elevated CRP (> 5mg/L)	-	-	149	60	-	-	17	71	-	-	104	61	-	-	11	65	-	-	110	62

CI, confidence interval; SBP, systolic blood pressure; DBP, diastolic blood pressure; BMI, body mass index; LSM, living standards measure; Hb, haemoglobin; Fer, serum ferritin; sTfR, soluble transferrin receptor; ID, iron deficiency; IDE, iron deficiency erythropoiesis; CRP, C-reactive protein; BMI categories: underweight: < 18.5 kg/m^2^, normal weight: 18.5 kg/m^2^–24.9 kg/m^2^, overweight: 25 kg/m^2^ – 29.9 kg/m^2^, obese: ≥30 kg/m^2^.

1 BMI data missing for 1 participant.

Women with SBP ≥ 120 mmHg in late pregnancy had a higher BMI at enrolment (29.6 [26.4, 35.4] kg/m^2^) compared to women with SBP < 120 mmHg (25.9 [25.0, 27.5] kg/m^2^). Anaemia prevalence (Hb < 10.5 g/dL) was 33% for women with SBP ≥ 120 mmHg and 16% for women with SBP < 120 mmHg. Anaemia prevalence using cut-offs of both 11 g/dL and 10.5 g/dL were higher in the higher DBP group (53% and 47%, respectively) compared to the lower DBP group (28% and 15%, respectively).

The characteristics of 54 (22%) participants without blood pressure data did not differ from the 196 (78%) participants with blood pressure data (Supplementary Table 1).

Table 2 shows that SBP, DBP and MAP were the lowest at mid-pregnancy, at 106.5 (± 9.6), 66.8 (± 7.9) and 80.9 (± 8.6) mmHg (*p* = 0.007, *p* < 0.001 and *p* = 0.001), respectively. Eighteen per cent of women had prehypertension at < 18 weeks’ gestation and remained similar during pregnancy. Heart rate increased over gestation (*p* < 0.001).

**TABLE 2:** Blood pressure of pregnant women at early, mid- and late pregnancy.

Blood pressure	< 18 weeks *n* = 248			± 22 weeks *n* = 234			± 36 weeks *n* = 196			*p*	*p*
	Mean ± s.d.	*n*	%	Mean ± s.d.	*n*	%	Mean ± s.d.	*n*	%		
SBP (mmHg)	108.3 ± 10.0^a^	-	-	106.5 ± 9.6^b^	-	-	108.6 ± 10.6^a^	-	-	0.007^1^	-
DBP (mmHg)	69.1 ± 8.3^a^	-	-	-	-	-	68.7 ± 8.3^a^	-	-	< 0.001^1^	-
Prehypertension	-	44	18	-	36	15	-	29	15	0.311^2^	0.327^2^
Hypertension	-	4	2	-	5	2	-	3	2	1.000^2^	1.000^2^
Heart rate (bpm)	82.0 ± 10.8^b^	-	-	86.3 ± 10.4^a^	-	-	87.6 ± 11.2^a^	-	-	< 0.001^1^	-
MAP (mmHg)	82.6 ± 8.5^a^	-	-	80.9 ± 8.6^b^	-	-	82.0 ± 8.5^a^	-	-	< 0.001^1^	-
eMAP	-	66	27	-	40	17	-	50	26	0.001^2^	< 0.001^2^

s.d., standard deviation; SBP, systolic blood pressure; DBP, diastolic blood pressure; MAP, mean arterial pressure; eMAP, elevated mean arterial pressure.

1 Longitudinal analysis of normal data was performed with the repeated measures ANOVA with Bonferroni corrections for between group analyses. Sphericity was assumed when *p* > 0.05 with Mauchly’s test. Greenhouse–Geisser was used when sphericity was violated. Means with different alphabetical letter superscripts in the same row differ significantly from each other.

2 Categorical associations between ± 22 and < 18 weeks as well as ± 36 and ±22 weeks were analysed with McNemar’s test for dependant data. *p* < 0.05 was considered significant and *p* between 0.05 and 0.08 had a tendency towards significance.

Fasting blood glucose at < 18 weeks’ gestation was 4.1 ± 0.4 mmol/L. After 120 min of consuming the glucose solution, blood glucose was 5.1 ± 1.2 mmol/L. Gestational diabetes mellitus was diagnosed in 4.3% of the women (results not shown).

Table 3a shows that the odds of prehypertension among women with anaemia (Hb < 10.5 g/dL) at ± 22 weeks’ gestation were three times (odds ratio [OR]: 3.01, 95% confidence interval [CI]: 1.22, 7.42) higher than the odds among women without anaemia as indicated in model 2. When additionally adjusted for blood pressure at mid-pregnancy, (Model 3) participants with anaemia (Hb < 10.5 g/dL) still tended to have 2.85 times higher odds for pregnancy-induced prehypertension than women without anaemia (OR: 2.85, 95% CI: 1.00, 8.10). Participants with anaemia (Hb < 10.5 g/dL) at < 18 weeks’ gestation tended to have 2.74 times higher odds for prehypertension than women without anaemia (OR: 2.74, 95% CI: 0.97, 7.80, *p* = 0.058).

**TABLE 3a:** Associations between maternal anaemia and iron status at early and mid-pregnancy with prehypertension in late pregnancy (multivariable logistic regression).

Gestational age	Predictor	Prehypertension (SBP ≥ 120 mmHg and/or DBP ≥ 80 mmHg)								
		Model 1 (*n* = 190)			Model 2 (*n* = 189)			Model 3		
		OR	95% CI	*p*	OR	95% CI	*p*	OR	95% CI	*p*
< 18 weeks	Anaemia (Hb < 10.5g/dL)	2.30	0.93, 5.70	***0.072***	2.74	0.97, 7.80	***0.058***	NA	-	-
	Anaemia (Hb < 11.0g/dL)	1.20	0.51, 2.78	0.680	1.42	0.55, 3.70	0.471	-	-	-
	ID (Fer < 15μg/L)	0.42	0.09, 1.92	0.265	0.43	0.08, 2.23	0.313	-	-	-
	IDE (sTfR > 8.3mg/L)	1.46	0.54, 4.00	0.457	2.06	0.65, 6.53	0.219	-	-	-
		**Model 1 (*n* = 189)**			**Model 2 (*n* = 188)**			**Model 3 (*n* = 188)**		
22 weeks	Anaemia (Hb < 10.5g/dL)	2.72	1.20, 6.16	**0.016**	3.01	1.22, 7.42	**0.016**	2.85	1.00, 8.10	***0.050***
	Anaemia (Hb < 11.0g/dL)	1.69	0.75, 3.80	0.204	1.91	0.78, 4.66	0.157	1.64	0.59, 4.55	0.342
	ID (Fer < 15μg/L)	0.42	0.12, 1.48	0.176	1.05	0.96, 1.15	0.248	0.42	0.08, 2.30	0.320
	IDE (sTfR > 8.3mg/L)	0.79	0.30, 2.11	0.644	0.72	0.26, 2.01	0.527	0.79	0.25, 2.53	0.692

OR, odds ratio; CI, confidence interval; Hb, haemoglobin; Fer, serum ferritin; sTfR, soluble transferrin receptor; bpm, beats per minute; ID, iron deficiency; IDE, iron deficiency erythropoiesis. Associations were assessed with multivariable logistic regression, odds ratios and 95% confidence intervals. Model 1 is adjusted for maternal age and living standards measure (socio-economic status); model 2 is adjusted additionally for gestational age, CRP and maternal BMI at baseline, HIV status throughout pregnancy, and aspirin prescription during mid-pregnancy; model 3 is adjusted additionally for blood pressure at ± 22 weeks’ gestation. *p* < 0.05 was considered significant and *p* between 0.05 and 0.08 had a tendency towards significance.

Participants with anaemia (Hb < 10.5 g/dL) at < 18 weeks’ gestation had 2.79 times higher odds for having elevated MAP than women without anaemia (OR: 2.79, 95% CI: 1.15, 6.79) in model 2. Participants with anaemia (Hb < 10.5 g/dL) at ± 22 weeks’ gestation had 2.15 times higher odds of having elevated MAP than women without anaemia (OR: 2.15, 95% CI: 1.01 4.60) in model 2 (Table 3b).

**TABLE 3b:** Associations between maternal anaemia and iron status at early and mid-pregnancy with elevated MAP in late pregnancy (multivariable logistic regression).

Gestational age	Predictor	Elevated MAP (MAP > 86 mmHg)								
		Model 1 (*n* = 189)			Model 2 (*n* = 184)			Model 3		
		OR	95% CI	*p*	OR	95% CI	*p*	OR	95% CI	*p*
< 18 weeks	Anaemia (Hb < 10.5g/dL)	1.91	0.87, 4.22	0.109	2.79	1.15, 6.79	**0.024**	NA	-	-
	Anaemia (Hb < 11.0g/dL)	0.94	0.46, 1.90	0.852	1.33	0.60, 2.95	0.490	-	-	-
	ID (Fer < 15μg/L)	0.67	0.24, 1.89	0.449	0.68	0.20, 2.24	0.523	-	-	-
	IDE (sTfR > 8.3mg/L)	1.56	0.67, 3.63	0.302	1.99	0.77, 5.13	0.157	-	-	-
		**Model 1 (*n* = 189)**			**Model 2 (*n* = 186)**			**Model 3 (*n* = 186)**		
22 weeks	Anaemia (Hb < 10.5g/dL)	1.89	0.96, 3.73	***0.067***	2.15	1.01, 4.60	**0.047**	1.92	0.77, 4.76	0.161
	Anaemia (Hb < 11.0g/dL)	1.38	0.71, 2.66	0.341	1.63	0.78, 3.38	0.191	1.65	0.68, 4.00	0.266
	ID (Fer < 15μg/L)	0.48	0.19, 1.24	0.127	0.55	0.20, 1.53	0.254	0.65	0.19, 2.15	0.477
	IDE (sTfR > 8.3mg/L)	0.74	0.33, 1.64	0.455	0.67	0.29, 1.59	0.368	0.61	0.23, 1.66	0.336

OR, odds ratio; CI, confidence interval; Hb, haemoglobin; Fer, serum ferritin; sTfR, soluble transferrin receptor; bpm, beats per minute; ID, iron deficiency; IDE, iron deficiency erythropoiesis; MAP, mean arterial pressure. Associations were assessed with multivariable logistic regression, odds ratios and 95% confidence intervals. Model 1 is adjusted for maternal age and living standards measure (socio-economic status); model 2 is adjusted additionally for gestational age, CRP and maternal BMI at baseline, HIV status throughout pregnancy, and aspirin prescription during mid-pregnancy; model 3 is adjusted additionally for blood pressure at ± 22 weeks’ gestation. *p* < 0.05 was considered significant and *p* between 0.05 and 0.08 had a tendency towards significance.

**TABLE 3c:** Associations between maternal anaemia and iron status at early and mid-pregnancy with heart rate in late pregnancy (multivariable logistic regression).

Gestational age	Predictor	Heart rate above median (HR > 87.5 bpm)								
		Model 1 (*n* = 189)			Model 2 (*n* = 184)			Model 3*		
		OR	95% CI	*p*	OR	95% CI	*p*	OR	95% CI	*p*
< 18 weeks	Anaemia (Hb < 10.5g/dL)	0.86	0.41, 1.81	0.681	1.02	0.47, 2.25	0.955	NA	-	-
	Anaemia (Hb < 11.0g/dL)	0.65	0.35, 1.22	0.182	0.77	0.40, 1.49	0.437	-	-	-
	ID (Fer < 15μg/L)	0.89	0.39, 2.02	0.784	0.80	0.34, 1.89	0.608	-	-	-
	IDE (sTfR > 8.3mg/L)	1.07	0.49, 2.31	0.869	1.28	0.57, 2.89	0.552	-	-	-
		**Model 1 (*n* = 189)**			**Model 2 (*n* = 186)**			-		
22 weeks	Anaemia (Hb < 10.5g/dL)	1.46	0.78, 2.74	0.233	1.39	0.72, 2.68	0.327	NA	-	-
	Anaemia (Hb < 11.0g/dL)	1.31	0.73, 2.34	0.370	1.37	0.74, 2.53	0.314	-	-	-
	ID (Fer < 15μg/L)	1.03	0.50, 2.13	0.942	1.10	0.52, 2.33	0.795	-	-	-
	IDE (sTfR > 8.3mg/L)	0.85	0.44, 1.67	0.639	0.74	0.37, 1.49	0.398	-	-	-

OR, odds ratio; CI, confidence interval; Hb, haemoglobin; Fer, serum ferritin; sTfR, soluble transferrin receptor; bpm, beats per minute; ID, iron deficiency; IDE, iron deficiency erythropoiesis; HR, heart rate. Associations were assessed with multivariable logistic regression, odds ratios and 95% confidence intervals. Model 1 is adjusted for maternal age and living standards measure (socio-economic status); model 2 is adjusted additionally for gestational age, CRP and maternal BMI at baseline, HIV status throughout pregnancy, and aspirin prescription during mid-pregnancy;

* , model 3, not performed. *p* < 0.05 was considered significant and *p* between 0.05 and 0.08 had a tendency towards significance.

### Associations of anaemia and iron status with blood pressure

Table 4a and 4b shows the results from the multivariable linear regression analyses on the associations of iron status at enrolment and mid-pregnancy with SBP and DBP (in mmHg) as outcomes, respectively. In model 2, Hb at ± 22 weeks’ gestation was associated negatively with SBP in late pregnancy (B = −1.10; 95% CI: -2.11, -0.09, *p* = 0.033). When additionally adjusting for blood pressure at mid-pregnancy (model 3), Hb at ± 22 weeks’ gestation tended to negatively associate with SBP in late pregnancy (B = −0.75; 95% CI: −1.59, 0.09, *p* = 0.081). In model 2, Hb at < 18 weeks’ gestation tended to associate negatively with SBP in late pregnancy (B = −0.87; 95% CI: −1.87, 0.13, *p* = 0.089). Similarly, in model 2, Hb at < 18 weeks’ gestation tended to associate negatively with DBP in late pregnancy (B = −0.72; 95% CI: −1.51, 0.07, *p* = 0.072). At ± 22 weeks’ gestation in model 3, Hb tended to associate negatively with DBP (B = −0.62; 95% CI: −1.31, 0.06, *p* = 0.074). Ferritin and sTfR at early and mid-pregnancy were not associated with blood pressure late in pregnancy.

**TABLE 4a:** Associations between maternal iron status at early and mid-pregnancy with systolic blood pressure in late pregnancy (multivariable linear regression).

Gestational age	Predictor	Systolic blood pressure (mmHg)								
		Model 1 (*n* = 190)			Model 2 (*n* = 189)			Model 3		
		B	95% CI	*p*	B	95% CI	*p*	B	95% CI	*p*
< 18 weeks	Hb (g/dL)	−0.46	−1.46, 0.53	0.358	−0.87	−1.87, 0.13	0.089	-	-	-
	Fer (μg/L)	−0.01	−0.02, 0.01	0.376	−0.01	−0.02, 0.01	0.273	-	-	-
	sTfR (mg/L)	0.04	−0.15, 0.22	0.709	0.08	−0.10, 0.26	0.407	-	-	-
		**Model 1 (*n* = 189)**			**Model 2 (*n* = 188)**			**Model 3 (*n* = 188)**		
22 weeks	Hb (g/dL)	−1.04	−2.06, -0.02	**0.046**	−1.10	−2.11, -0.09	**0.033**	−0.75	−1.59, 0.09	***0.081***
	Fer (μg/L)	−0.01	−0.04, 0.02	0.660	−0.02	−0.04, 0.01	0.310	−0.01	−0.03, 0.02	0.695
	sTfR (mg/L)	−0.03	−0.33, 0.27	0.858	−0.03	−0.32, 0.28	0.831	−0.07	−0.31, 0.17	0.566

CI, confidence interval; Hb, haemoglobin; Fer, serum ferritin; sTfR, soluble transferrin receptor. Associations were assessed with multivariable linear regression, B-values and 95% confidence intervals. Model 1 is adjusted for maternal age and living standards measure (socio-economic status); model 2 is adjusted additionally for gestational age, CRP and maternal BMI at baseline, HIV status throughout pregnancy and aspirin prescription during mid-pregnancy; model 3 is adjusted additionally for blood pressure at ± 22 weeks’ gestation. *p* < 0.05 was considered significant and *p* between 0.05 and 0.08 had a tendency towards significance.

**TABLE 4b:** Associations between maternal iron status at early and mid-pregnancy with diastolic blood pressure in late pregnancy (multivariable linear regression).

Gestational age	Predictor	Diastolic blood pressure (mmHg)								
		Model 1 (*n* = 190)			Model 2 (*n* = 189)			Model 3		
		B	95% CI	*p*	B	95% CI	*p*	B	95% CI	*p*
< 18 weeks	Hb (g/dL)	−0.47	−1.23, 0.30	0.232	−0.72	−1.51, 0.07	***0.072***	-	-	-
	Fer (μg/L)	0.00	−0.01, 0.01	0.752	0.00	−0.01, 0.01	0.745	-	-	-
	sTfR (mg/L)	0.02	−0.12, 0.17	0.766	0.05	−0.09, 0.19	0.465	-	-	-
		**Model 1 (*n* = 189)**			**Model 2 (*n* = 188)**			**Model 3 (*n* = 188)**		
22 weeks	Hb (g/dL)	−0.60	−1.40, 0.19	0.136	−0.59	−1.39, 0.21	0.146	−0.62	−1.31, 0.06	***0.074***
	Fer (μg/L)	0.00	−0.02, 0.02	0.987	−0.01	−0.03, 0.02	0.633	−0.01	−0.03, 0.01	0.422
	sTfR (mg/L)	−0.01	−0.24, 0.22	0.938	−0.02	−0.25, 0.21	0.865	−0.02	−0.21, 0.18	0.866

CI, confidence interval; Hb, haemoglobin; Fer, serum ferritin; sTfR, soluble transferrin receptor. Associations were assessed with multivariable linear regression, B-values and 95% confidence intervals. Model 1 is adjusted for maternal age and living standards measure (socio-economic status); model 2 is adjusted additionally for gestational age, CRP and maternal BMI at baseline, HIV status throughout pregnancy and aspirin prescription during mid-pregnancy; model 3 is adjusted additionally for blood pressure at ± 22 weeks’ gestation. *p* < 0.05 was considered significant and *p* between 0.05 and 0.08 had a tendency towards significance.

In Table 4c, model 2, Hb at < 18 weeks’ gestation tended to associate negatively with MAP (B = −0.77; 95% CI: -1.58, 0.04, *p* = 0.063). At ± 22 weeks’ gestation at models 2 and 3, Hb tended to associate negatively with MAP in late pregnancy (B = −0.76; 95% CI: −1.59, 0.06, *p* = 0.070; B = -0.67; 95% CI: −1.36, 0.02, *p* = 0.056, respectively). Ferritin and sTfR at early and mid-pregnancy were not associated with MAP during late pregnancy. Table 4d shows that Ferritin at < 18 weeks’ gestation was negatively associated with heart rate in late pregnancy (B = −0.02; 95% CI: −0.03, 0.00, *p* = 0.041). Hb and sTfR at early and mid-pregnancy were not associated with heart rate in late pregnancy.

**TABLE 4c:** Associations between maternal iron status at early and mid-pregnancy with mean arterial pressure in late pregnancy (multivariable linear regression).

Gestational age	Predictor	MAP (mmHg)								
		Model 1 (*n* = 189)			Model 2 (*n* = 184)			Model 3		
		B	95% CI	*p*	B	95% CI	*p*	B	95% CI	*p*
< 18 weeks	Hb (g/dL)	−0.47	−1.27, 0.34	0.253	−0.77	−1.58, 0.04	***0.063***	-	-	-
	Fer (μg/L)	0.00	−0.02, 0.01	0.570	0.00	−0.02, 0.01	0.509	-	-	-
	sTfR (mg/L)	0.03	−0.12, 0.18	0.730	0.06	−0.09, 0.21	0.416	-	-	-
		**Model 1 (*n* = 189)**			**Model 2 (*n* = 186)**			**Model 3 (*n* = 186)**		
22 weeks	Hb (g/dL)	−0.75	−1.58, 0.08	***0.075***	−0.76	−1.59, 0.06	***0.070***	−0.67	−1.36, 0.02	***0.056***
	Fer (μg/L)	0.00	−0.03, 0.02	0.865	−0.01	−0.03, 0.01	0.469	−0.01	−0.03, 0.01	0.468
	sTfR (mg/L)	−0.02	−0.26, 0.23	0.901	−0.02	−0.26, 0.21	0.843	−0.03	−0.23, 0.16	0.736

CI, confidence interval; Hb, haemoglobin; Fer, serum ferritin; sTfR, soluble transferrin receptor. Associations were assessed with multivariable linear regression, B-values and 95% confidence intervals. Model 1 is adjusted for maternal age and living standards measure (socio-economic status); model 2 is adjusted additionally for gestational age, CRP and maternal BMI at baseline, HIV status throughout pregnancy and aspirin prescription during mid-pregnancy; model 3 is adjusted additionally for blood pressure at ± 22 weeks’ gestation. *p* < 0.05 was considered significant and *p* between 0.05 and 0.08 had a tendency towards significance.

**TABLE 4d:** Associations between maternal iron status at early and mid-pregnancy with heart rate in late pregnancy (multivariable linear regression).

Gestational age	Predictor	Heart rate (bpm)								
		Model 1 (*n* = 189)			Model 2 (*n* = 184)			Model 3*		
		B	95% CI	*p*	B	95% CI	*p*	B	95% CI	*p*
< 18 weeks	Hb (g/dL)	0.56	−0.50, 1.62	0.295	0.45	−0.66, 1.55	0.425	-	-	-
	Fer (μg/L)	−0.02	−0.03, 0.00	**0.023**	−0.02	−0.03, 0.00	**0.041**	-	-	-
	sTfR (mg/L)	0.09	−0.11, 0.29	0.364	0.11	−0.08, 0.31	0.254	-	-	-
		**Model 1 (*n* = 189)**			**Model 2 (*n* = 186)**			-		
22 weeks	Hb (g/dL)	−0.32	−1.41, 0.77	0.568	−0.21	−1.31, 0.89	0.710	-	-	-
	Fer (μg/L)	−0.03	−0.06, 0.01	0.112	−0.02	−0.05, 0.01	0.123	-	-	-
	sTfR (mg/L)	0.08	−0.24, 0.39	0.640	0.07	−0.24, 0.38	0.664	-	-	-

CI, confidence interval; Hb, haemoglobin; Fer, serum ferritin; sTfR, soluble transferrin receptor. Associations were assessed with multivariable linear regression, B-values and 95% confidence intervals. Model 1 is adjusted for maternal age and living standards measure (socio-economic status); model 2 is adjusted additionally for gestational age, CRP and maternal BMI at baseline, HIV status throughout pregnancy and aspirin prescription during mid-pregnancy;

* , model 3, not conducted. *p* < 0.05 was considered significant and *p* between 0.05 and 0.08 had a tendency towards significance.

Supplementary Table 2 shows that women in the lowest Hb quartile (Hb < 10.8 g/dL) at ± 22 weeks of gestation had a higher SBP in late pregnancy by 4.16 mmHg (B = 4.16, 95% CI: 0.41, 7.92; *p* = 0.030) and a higher DBP by 4.08 mmHg (B = 4.08; 95% CI: 1.05, 7.12; *p* = 0.009) when compared to women in the highest Hb quartile (Hb > 12.1g/dL). There were no associations for Hb, ferritin or sTfR at < 18 weeks of gestation with SBP or DBP in late pregnancy.

### Associations of iron status biomarkers with glucose tolerance

No associations were found between any of the iron status markers and glucose tolerance at mid-pregnancy (Supplementary Table 3).

## Discussion

The authors found that pregnant women with anaemia (Hb < 10.5 g/dL) in early and mid-pregnancy had almost three times higher odds of presenting with prehypertension and double the odds for elevated MAP in late pregnancy in this urban population, despite routine iron supplementation.

Similarly, a large global study (*n* = 214 067) by Chen et al. (2018) found that women with severe anaemia (Hb < 7 g/dL) had an increased odds of gestational hypertension and preeclampsia or eclampsia. Thus, even though the clinical significance in this study of one unit decrease in Hb predicting an increase in SBP with 1.1 mmHg seems minimal, this larger study provides evidence of a sliding scale relationship with worsening anaemia. In countries struggling to manage gestational hypertension, such as South Africa, the association the authors found between anaemia and higher blood pressure may be especially important. Hypertensive disorders of pregnancy account for 18% of maternal deaths (Ngene & Daef 2021). In this study’s sample of pregnant women, 18%, 15% and 15% of women were prehypertensive at early, mid- and late pregnancy, respectively, despite routine iron, folic acid and calcium supplementation. These women also received aspirin treatment at mid-pregnancy when indicated, to prevent or delay the onset of preeclampsia (Davidson et al. 2021). Thus, if anaemia is to be considered a predictor of higher blood pressure (BP), treating anaemia effectively will contribute significantly to reducing morbidity related to gestational hypertension. The anaemia-BP relationship at late pregnancy was independent of blood pressure at mid-pregnancy and diagnosed hypertension was excluded, suggesting that this association is largely precipitated by pregnancy. The authors did not find a U-shaped relationship (Young et al. 2019), likely because no women had high Hb values.

The anaemia-BP relationship may be related to low plasma volume expansion, but the mechanism is still hypothetical (Aguree & Gernand 2019). Low plasma volume is thought to negatively affect cardiac preload resulting in a compensation of the sympathetic nervous system to the reduced venous reserve and consequently leading to increased shear stress on the vascular endothelium (Scholten et al. 2015). Another hypothesised mechanism is that anaemia-induced hypoxia may be related to preeclampsia as it causes increased heart rate and cardiac output via β-adrenergic stimulation (Faulhaber et al. 2015). This research also confirmed the current dogma that there is a mid-trimester drop in BP (Shen et al. 2017).

Along with this study’s findings of increased blood pressure, ferritin in early pregnancy showed a small negative association with heart rate in late pregnancy. In congruence, others have found that IDA along with vasomotor changes resulted in increased heart rate (Faulhaber et al. 2015). This is clinically significant if ferritin can be increased sufficiently as may happen with effective treatment of ID during pregnancy. This may be important as anaemia has been associated with an exacerbated increase in heart rate which can increase the risk for cardiac disease and maternal morbidity (Coad & Frise 2021).

Lastly, the authors found no associations between any of the iron status markers and glucose tolerance at mid-pregnancy. In agreement with this study, a meta-analysis by Kataria et al. (2018) found no association between iron overload and GDM and suggested the need for a systematic review to assess the relationship between iron biomarkers and GDM. This study’s findings differed from others who found associations with blood glucose and either elevated or decreased Hb (Wang et al. 2018; Young et al. 2019). Current research regarding the association between iron supplementation and GDM is contradictory, and the extent to which the risk of GDM is affected appears relatively small (Petry 2022).

Strengths of this study include prospective data with multiple variables and over the course of pregnancy. In addition, multiple iron biomarkers were assessed, giving a complete description of iron status. This study’s analyses also included CRP and AGP, which were used to adjust ferritin concentrations. This study included several confounders which strengthened its analyses. The study limitations should be considered when interpreting the results. The sample was relatively small and not representative of the general population. Furthermore, it should be considered that women who delivered prematurely (11%) and therefore missed their 36 weeks’ gestation visit (Symington et al. 2019) may have had complications due to increased blood pressure but could not have been accounted for in this analysis. Lastly, there was a large variability in gestational age particularly at enrolment (about 7–18 weeks’ gestation), which would affect the analyses during early pregnancy. This was statistically corrected.

## Conclusion

In conclusion, the authors found that anaemia at mid-pregnancy was associated with three times higher odds of being prehypertensive and double the odds of having elevated MAP at late pregnancy in women residing in urban South Africa. This is despite routine iron (as well as folic acid and calcium) supplementation. The challenge remains how to effectively manage anaemia in pregnant women and ultimately reduce maternal morbidity.
